# Supplementary material for: Lack of Retinoblastoma Protein Shifts Tumor Metabolism from Glycolysis to OXPHOS and Allows the Use of Alternate Fuels
Source: Cells. 2022 Oct 11;11(20):3182. doi: 10.3390/cells11203182 (PMC9600484; doi:10.3390/cells11203182)
Supplement: Supplementary file 1 [file cells-11-03182-s001.zip › cells-1869491-supplementary.pdf]

Manuscript ID: Cells-1869491

Supplementary Figures

Figure S1

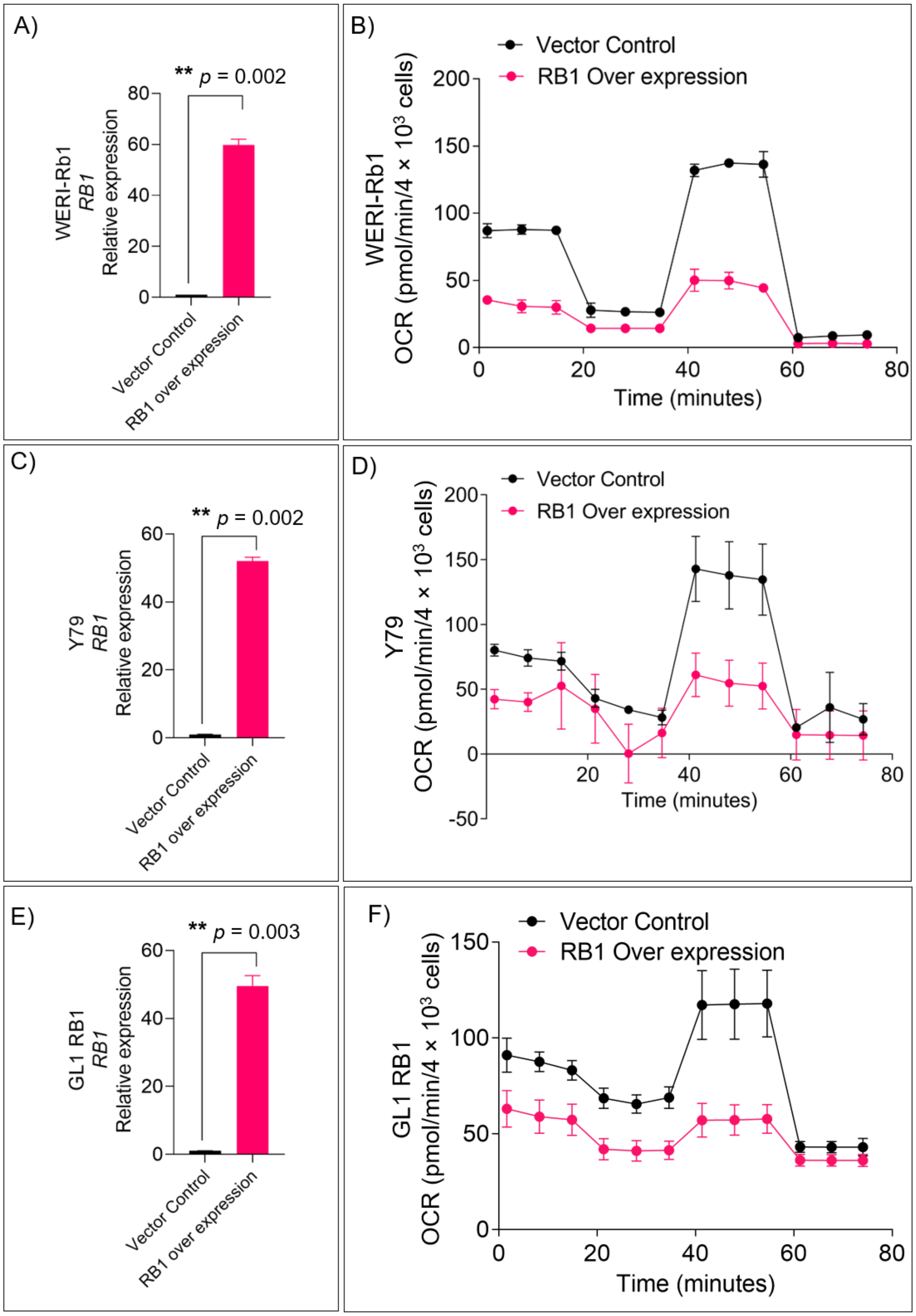

Figure S1. RB1 expression halts mitochondrial respiration in retinoblastoma cells. A) RT-PCR showing *RB1* expression in control and *RB1* complemented WERI-Rb1 cells. B) Seahorse XF Mito-stress assay showing mitochondrial respiration profile in control and *RB1* complemented WERI-Rb1 cells. C) RT-PCR showing *RB1* expression in control and *RB1* complemented Y79 cells. D) Seahorse XF Mito-stress assay showing mitochondrial respiration profile in control and *RB1* complemented Y79 cells. E) RT-PCR showing *RB1* expression in control and *RB1* complemented GL1-RB1 cells. F) Seahorse XF Mito-stress assay showing mitochondrial respiration profile in control and *RB1* complemented GL1-RB1 cells. Values represent three independent experiments mean with data points ± S.E.M. Two-tailed Mann-Whitney test was used for statistical analysis. * $p < 0.05$, ** $p < 0.01$.

Figure S2:

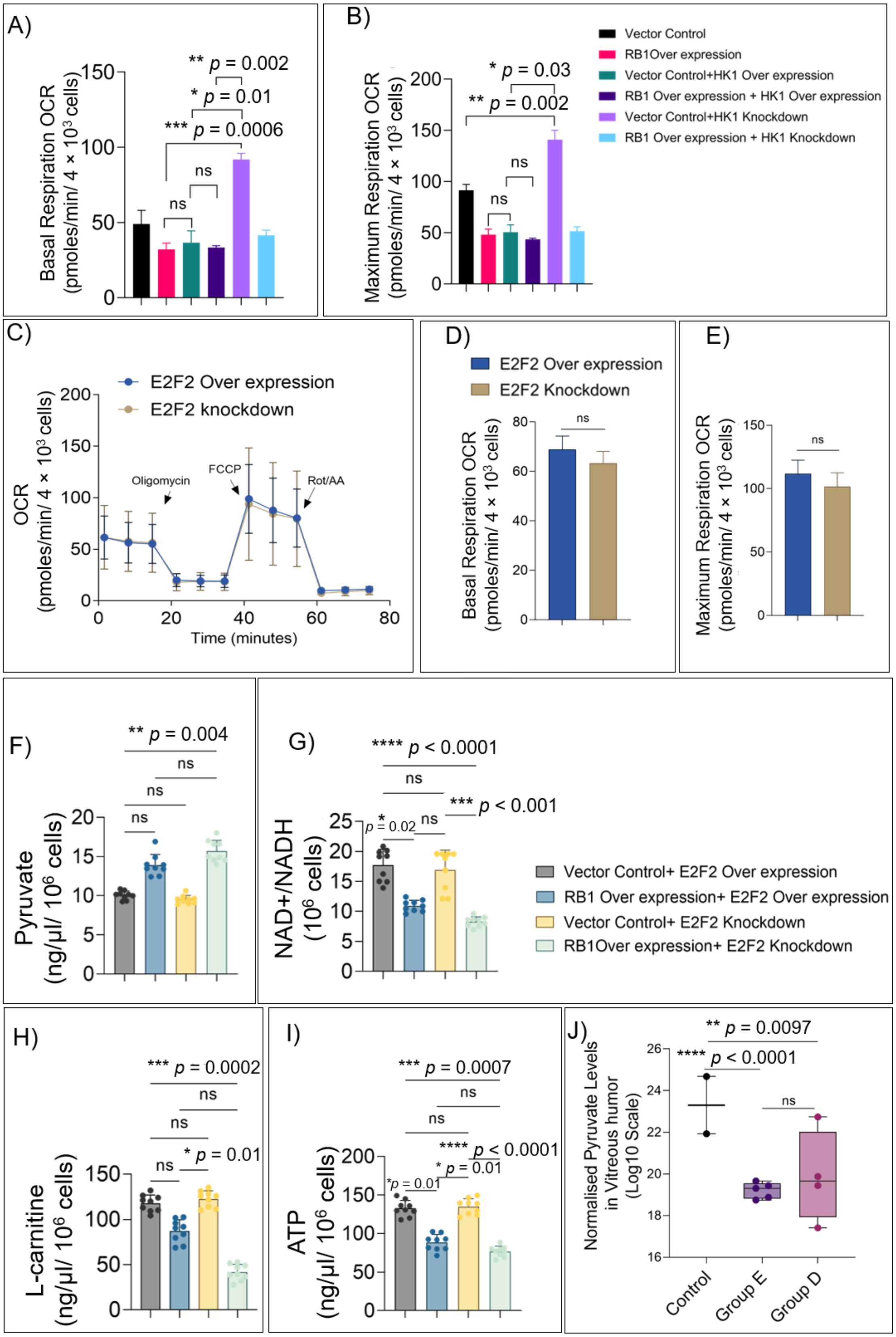

A)
Basal Respiration OCR (pmoles/min/ 4 × 10³ cells)
** p = 0.002
* p = 0.01
*** p = 0.0006
ns
ns
B)
Maximum Respiration OCR (pmoles/min/ 4 × 10³ cells)
* p = 0.03
** p = 0.002
ns
ns
Vector Control
RB1Over expression
Vector Control+HK1 Over expression
RB1 Over expression + HK1 Over expression
Vector Control+HK1 Knockdown
RB1 Over expression + HK1 Knockdown
C)
E2F2 Over expression
E2F2 knockdown
OCR (pmoles/min/ 4 × 10³ cells)
Oligomycin
FCCP
Rot/AA
Time (minutes)
D)
E2F2 Over expression
E2F2 Knockdown
Basal Respiration OCR (pmoles/min/ 4 × 10³ cells)
ns
E)
Maximum Respiration OCR (pmoles/min/ 4 × 10³ cells)
ns
F)
** p = 0.004
ns
ns
ns
Pyruvate (ng/µl/ 10⁶ cells)
G)
**** p < 0.0001
ns
*** p < 0.001
* p = 0.02
ns
NAD+/NADH (10⁶ cells)
Vector Control+ E2F2 Over expression
RB1 Over expression+ E2F2 Over expression
Vector Control+ E2F2 Knockdown
RB1Over expression+ E2F2 Knockdown
H)
*** p = 0.0002
ns
ns
ns
* p = 0.01
L-carnitine (ng/µl/ 10⁶ cells)
I)
*** p = 0.0007
ns
ns
**** p < 0.0001
* p = 0.01
*p = 0.01
ATP (ng/µl/ 10⁶ cells)
J)
** p = 0.0097
**** p < 0.0001
ns
Normalised Pyruvate Levels in Vitreous humor (Log10 Scale)
Control
Group E
Group D

Figure S2. HK1 and RB1 expression induce a metabolic switch from mitochondrial respiration to glycolysis. WERI-Rb1 cells were transduced with indicated vectors modulating RB1 and HK1 as indicated and oxygen consumption rate (OCR) was measured using Seahorse XFp Mito-stress assay to assess mitochondrial function in WERI-Rb1 cells, A) Basal respiration, B) Maximum respiration. C) WERI-Rb1 cells were transduced with indicated vectors modulating E2F2 OE and E2F2 KD as indicated and oxygen consumption rate was measured using Seahorse XFp Mito-stress assay to assess mitochondrial function (n=1). D) Basal respiration, E) Maximum respiration. In cells transduced with indicated vectors, measurement of key metabolites – F) Pyruvate levels indicating glycolytic flux G) NAD+/NADH ratios to estimate mitochondrial TCA functions H) L-carnitine levels to evaluate dependency on fatty acid oxidation I) Total ATP levels to estimate energy produced. J) Pyruvate levels measured in Rb Group E vitreous humor (n=5) compared to Group D (n=4) and pediatric subjects (n=2) using LC-MS. Two-tailed Mann-Whitney test (for 2 groups) or Kruskal–Wallis with Dunn's multiple comparisons test (for >2 groups) were used for statistical analysis. * $p < 0.05$, ** $p < 0.01$, *** $p < 0.001$, **** $p < 0.0001$

Figure S3:

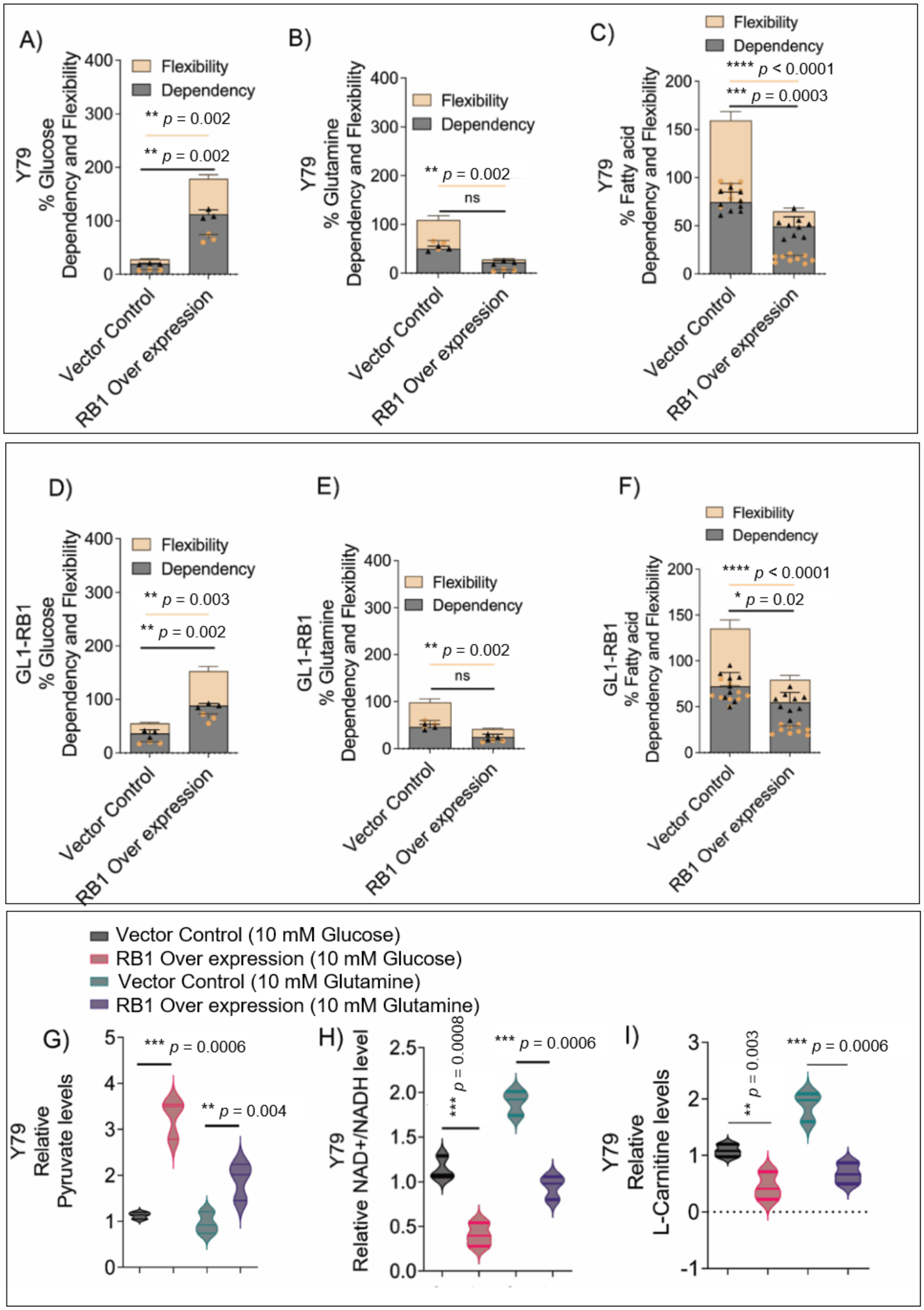

Figure S3: RB1 expression restricts the usage of alternate fuels. Seahorse XFp Mito-fuel flex assays to measure fuel dependency and flexibility in cells transduced with different vectors as indicated (A) Oxidation of glucose as fuel in control and RB1 complemented Y79 cells. (B) Oxidation of glutamine as fuel in control and RB1 complemented Y79

cells. (C) Oxidation of fatty acids as fuel in control and RB1 complemented Y79 cells (n=3). Validation of fuel oxidation profile in cells cultured in glucose and glutamine alone conditions and transduced with indicated vectors, measurement of key metabolites – (D) Oxidation of glucose as fuel in control and RB1 complemented GL1-RB1 cells. (E) Oxidation of glutamine as fuel in control and RB1 complemented GL1-RB1 cells. (F) Oxidation of fatty acids as fuel in control and RB1 complemented GL1-RB1 cells (n=3). (G) Pyruvate levels indicating glycolytic flux in Y79 cells (H) NAD+/NADH ratios to estimate mitochondrial TCA functions in Y79 cells (I) L-carnitine levels to evaluate dependency on fatty acid oxidation in Y79 cells. Values represent the mean ± s.d of three independent experiments. Two-tailed Mann-Whitney test (for 2 groups) or Kruskal–Wallis with Dunn's multiple comparisons test (for >2 groups) were used for statistical analysis. *$p < 0.05$, **$p < 0.01$, *** $p < 0.001$, **** $p < 0.0001$.

Figure S4

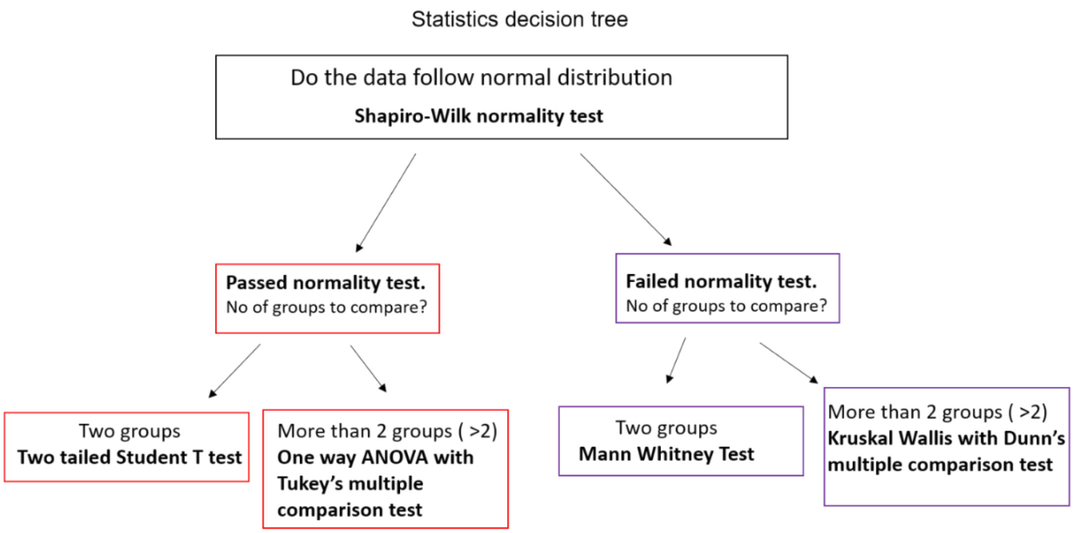

Figure S4. Statistical decision tree

Table S1: Details of lentiviral plasmids used in the study:

Plasmid	Cat#	Sequence	3'	5'	Loop
HK1 shRNA Rank1	ULTRA-3297147	TGCTGTTGACAGTGAGCGACTGCTACATGGAGGAGATGAAT AGTGAAGCCACAGATGTATTCATCTCCTCCATGTAGCAGGT GCCTACTGCCTCGGA	TTCATCTCCTCCATGTAGCAGG	ACTGCTACATGGAGGAGATGAA	TAGTGAAGCCACAGATGTA
HK1 shRNA Rank2	ULTRA-3297148	TGCTGTTGACAGTGAGCGATCCGATGAAACTCTCATAGAAT AGTGAAGCCACAGATGTATTCTATGAGAGTTTCATCGGAGT GCCTACTGCCTCGGA	TTCTATGAGAGTTTCATCGGAG	ATCCGATGAAACTCTCATAGAA	TAGTGAAGCCACAGATGTA
HK1 shRNA Rank2	ULTRA-3297150	TGCTGTTGACAGTGAGCGCCAAGGACAAGAAGTTACCTGAT AGTGAAGCCACAGATGTATCAGGTAACTTCTTGTCCTTGAT GCCTACTGCCTCGGA	TCAGGTAACTTCTTGTCCTTGA	CCAAGGACAAGAAGTTACCTGA	TAGTGAAGCCACAGATGTA
E2F2 shRNA Rank1	ULTRA-3231765	TGCTGTTGACAGTGAGCGACCCGGGGAGAAGACTCGGTATT AGTGAAGCCACAGATGTAATACCGAGTCTTCTCCCCGGGGT GCCTACTGCCTCGGA	ATACCGAGTCTTCTCCCCGGGG	ACCCGGGGAGAAGACTCGGTAT	TAGTGAAGCCACAGATGTA
E2F2 shRNA Rank2	ULTRA-3231766	TGCTGTTGACAGTGAGCGATGAGGACAACCTGCAGATATAT AGTGAAGCCACAGATGTATATATCTGCAGGTTGTCCTCAGT GCCTACTGCCTCGGA	TATATCTGCAGGTTGTCCTCAG	ATGAGGACAACCTGCAGATATA	TAGTGAAGCCACAGATGTA
E2F2 shRNA Rank3	ULTRA-3231767	TGCTGTTGACAGTGAGCGCCAGCGATCTCTTCGACTCCTAT AGTGAAGCCACAGATGTATAGGAGTCGAAGAGATCGCTGAT GCCTACTGCCTCGGA	TAGGAGTCGAAGAGATCGCTGA	CCAGCGATCTCTTCGACTCCTA	TAGTGAAGCCACAGATGTA
Non Target shRNA control	ULTRA-NT#4	TGCTGTTGACAGTGAGCGaaggcagaagtatgcaaagcatT AGTGAAGCCACAGATGTAatgctttgcatacttctgcctgT GCCTACTGCCTCGGA	atgctttgcatacttctgcctg	aaggcagaagtatgcaaagcat	TAGTGAAGCCACAGATGTA
